# PhosphoDisco: A Toolkit for Co-regulated Phosphorylation Module Discovery in Phosphoproteomic Data

**DOI:** 10.1016/j.mcpro.2023.100596

**Published:** 2023-06-30

**Authors:** Tobias Schraink, Lili Blumenberg, Grant Hussey, Sabrina George, Brecca Miller, Nithu Mathew, Tania J. González-Robles, Vladislav Sviderskiy, Thales Papagiannakopoulos, Richard Possemato, David Fenyö, Kelly V. Ruggles

**Affiliations:** 1 Division of Precision Medicine, Department of Medicine, New York University Grossman School of Medicine, New York, New York, USA; 2 Institute for Systems Genetics, New York University Grossman School of Medicine, New York, New York, USA; 3 Department of Biochemistry and Molecular Pharmacology, New York University Grossman School of Medicine, New York, New York, USA; 4 Department of Pathology, New York University Grossman School of Medicine, New York, New York, USA

**Keywords:** phosphoproteomics, co-regulation, modules, cancer, tandem mass spectrometry

## Abstract

Kinases are key players in cancer-relevant pathways and are the targets of many successful precision cancer therapies. Phosphoproteomics is a powerful approach to study kinase activity and has been used increasingly for the characterization of tumor samples leading to the identification of novel chemotherapeutic targets and biomarkers. Finding co-regulated phosphorylation sites which represent potential kinase-substrate sets or members of the same signaling pathway allows us to harness these data to identify clinically relevant and targetable alterations in signaling cascades. Unfortunately, studies have found that databases of co-regulated phosphorylation sites are only experimentally supported in a small number of substrate sets. To address the inherent challenge of defining co-regulated phosphorylation modules relevant to a given dataset, we developed PhosphoDisco, a toolkit for determining co-regulated phosphorylation modules. We applied this approach to tandem mass spectrometry based phosphoproteomic data for breast and non-small cell lung cancer and identified canonical as well as putative new phosphorylation site modules. Our analysis identified several interesting modules in each cohort. Among these was a new cell cycle checkpoint module enriched in basal breast cancer samples and a module of PRKC isozymes putatively co-regulated by CDK12 in lung cancer. We demonstrate that modules defined by PhosphoDisco can be used to further personalized cancer treatment strategies by establishing active signaling pathways in a given patient tumor or set of tumors, and in providing new ways to classify tumors based on signaling activity.

## Highlights

• PhosphoDisco is a computational approach to define co-regulated phosphosites.
• Implementation finds signaling modules in breast and non-small cell lung cancer.
• Identified a cell cycle checkpoint module in enriched basal breast tumors.
• PRKC-associated and proliferative enriched modules found in lung tumors.

## Graphical Abstract

**Figure:**
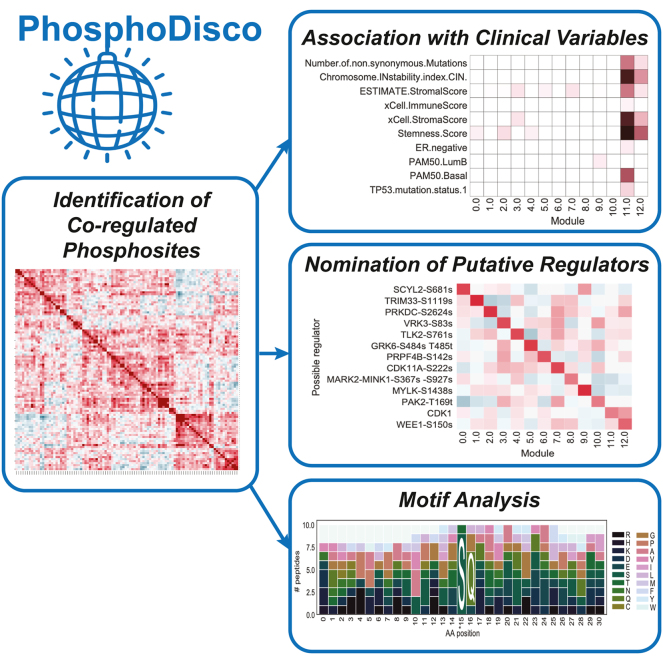

## In Brief

Schraink, Blumenberg, and Hussey *et al.*, demonstrate the use of PhosphoDisco, a newly developed tookit for the identification of co-regulated phosphorylation sites from tandem mass spectrometry–based phosphoproteomic data. Using data from breast and non-small cell lung cancers they identify several clinically relevant phosphorylation modules including a cell cycle checkpoint module enriched in basal breast cancer samples and a module of PRKC isozymes putatively co-regulated by CDK12 in lung cancer.

Protein phosphorylation results in conformational changes, leading to changes in protein activity, substrate affinity, and degradation. This process is regulated by enzymatic kinases and phosphatases that catalyze the transfer of phosphate between their substrates. Subsequently, the activity of these enzymes results in the activation or deactivation of signaling pathways that drive different cellular processes such as cell growth, apoptosis, and differentiation. Improper regulation of these pathways can lead to severe disease states such as cancer. Although protein kinase genes account for only 2% of human genes, up to 30% of all human proteins can be modified by kinase activity (1, 2). Therefore, exploring the role of phosphoproteins and the mechanisms of kinases is vital in contributing to our understanding of cancer biology. Further, kinases are eminently targetable and represent some of the most successful personalized cancer therapeutics developed to date (3, 4, 5, 6, 7).

Quantitation of the phosphoproteome by mass spectrometry (MS) provides a particularly useful perspective on signaling patterns and vulnerabilities in cancer and can provide a personalized view of aberrations in potentially targetable pathways. However, comprehensively discerning the activity of phosphorylation signaling pathways is challenging because phosphorylation is often context-dependent and significantly modified in cancer. To address this challenge, the Clinical Proteomics Tumor Analysis Consortium (CPTAC) has conducted proteogenomic characterization of several cancer types by performing DNA and RNA sequencing, as well as MS-based proteomic and phosphoproteomic analysis (8, 9, 10, 11, 12, 13, 14, 15, 16, 17). These efforts have generated rich high-dimensional data sets which have been used to generate and test novel hypotheses. However, identifying relevant pathways from these large-scale omics studies can be difficult, largely due to their high dimensionality and co-linearity. Further, it has been shown that the correlation between kinase abundance and known substrate sets in multiple CPTAC data sets is close to what would be expected by chance (18) and independently identified associations between phosphopeptides and kinases have minimal overlap with these curated sets (19). There are many reasons why kinase-substrate sets curated from myriad sources would not be reflected in a particular cancer dataset, but the challenge of defining co-regulated phosphosites in cancer data remains.

To better define these co-regulated modules in patient samples, we developed a computational toolkit, PhosphoDisco, for the analysis of tumor phosphoproteomic and proteomic data. We applied PhosphoDisco to data from a cohort of breast cancer (BRCA) tumors, and a combined data set of lung squamous cell carcinoma (LSCC) and lung adenocarcinoma (LUAD) tumors with their respective matched normal samples to define co-regulated phosphorylation modules within and across data sets. We show that putative modules can be used to nominate biomarkers for disease-specific treatments, as well as targets for novel treatment strategies, and highlight the strength of pan-cancer phosphoproteomics for cancer discovery.

## Experimental Procedures

### Proteomics and Phosphoproteomics Data

The dataset we chose to demonstrate the utility of PhosphoDisco was collected and processed as part of the CPTAC consortium. Comprehensive characterization of the BRCA (17), LSCC (15), and LUAD (16) tumor and matched normal cohorts (Table 1) have been completed, and all samples were collected and processed according to the CPTAC standard protocols (20). Detailed experimental procedures including cohort statistics, clinical data, sample collection and processing, and data acquisition for these cohorts are described in detail elsewhere (15, 16, 17).

**Table 1:** Dataset summary stats

Tumor type	Number of tumor samples	Number of normal samples	Number of phosphosites assigned to modules	Number of modules
LUAD	98	98	1684	14
LSCC	99	99	1684	14
BRCA	122	0	1017	69

Lists basic summary stats about the main data sets used in this paper. The two lung datasets (LSCC, LUAD) were used in a combined analysis, and included normal samples, while the breast dataset (BRCA) only included tumor samples.

Briefly, tumor samples were snap-frozen less than 30 min after collection, after which genomic and transcriptomic sequencing was completed. Samples also underwent higher-energy C-trap dissociation (HCD) liquid chromatography (LC)-MS/MS analysis of tandem mass tag (TMT)-labeled samples for proteomic and phosphoproteomic characterization as previously described (20).

### Data Processing and Quality Control

The Spectrum Mill software package v7.0 pre-release (Agilent Technologies, Santa Clara, CA) co-developed by Karl Clauser of the Carr laboratory (https://www.broadinstitute.org/proteomics) was used for MS data analysis. Protein identification was performed by searching the MS/MS spectra against the protein sequence database obtained using the UCSC Table Browser (https://genome.ucsc.edu/cgi-bin/hgTables) on September 14, 2016, which contains 37,579 proteins mapped to the human reference genome (hg19), adding common contaminants, mitochondrial proteins, and non-canonical small open reading frames. The searches were performed allowing ±20 ppm mass tolerance for precursor and product ions, allowing for common modification. Peptide spectrum matches (PSMs) were filtered for 30% minimum matched peak intensity and target-decoy-based false discovery rate (FDR) estimates at the PSM level, and for proteins protein level for each TMT-plex for all TMT-plexes for a tumor type, and for phosphorylation at the site levels. Normalization of each peptide was performed using the common reference, and a two-component Gaussian mixture model-based normalization was used to nullify the effect of differential protein loading and/or systematic MS variation.

### PhosphoDisco Workflow

**Fig. 1:**
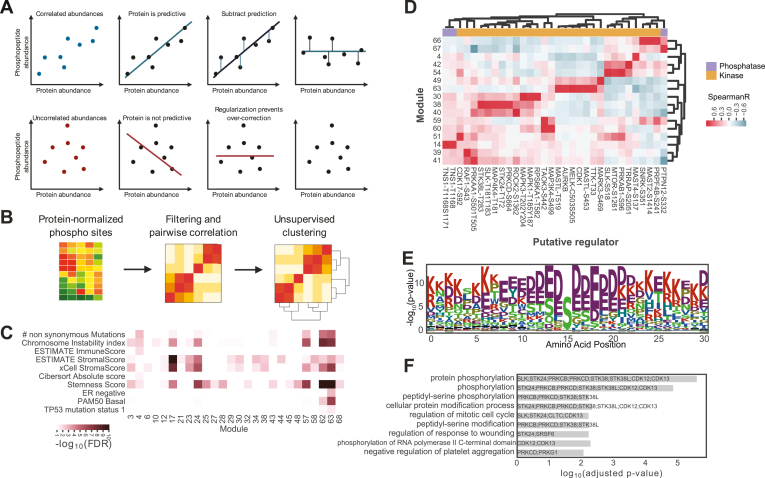
**PhosphoDisco core functionalities.***A*, regularized linear models are used to normalize phosphorylation data by protein abundance. *B*, normalized phosphorylation data are correlated with itself and modules are found by clustering the correlation matrix using hypercluster. *C*, clinical metadata are correlated with module scores to find relevant modules. *D*, kinase and phosphatase abundances are correlated with module scores and assigned potential regulators to modules. *E*, motifs can be calculated from peptides for a module. *F*, enrichments of phosphosites in a module can be calculated against a phosphosite annotations database like PhosphositePlus.

The PhosphoDisco workflow (supplemental Fig. S1 and supplemental Table S1) starts with normalizing, filtering, and pairwise correlation of peptide-level phosphorylation data, described in more detail below. The next steps are to find co-regulated modules relevant to clinical annotations, followed by nominating putative protein regulators for the modules. Further, Gene Ontology (GO) enrichment is then applied to find modules associated with annotated pathways, and motif analysis of phosphosite flanking sequences can identify common motifs across phosphosites within the module. Identification of kinase activation loop phosphosites, druggability analysis, and PTM set enrichment analysis is also applied to help rank nominated modules and their regulators (Fig. 1). Together, this approach enables the user to reduce complex phosphoproteomic data sets into potentially relevant signaling modules for further biological interrogation.

PhosphoDisco comes with a Snakemake (21) pipeline that can be run using phdc_run *via* the command line. Before running the pipeline, the user should generate a configuration file that includes the path to the phosphorylation and protein data files. Input and output structure, as well as example workflows can be found in the PhosphoDisco tutorial which comes with each PhosphoDisco installation. Although built to be run on a high-performance compute cluster, all the computation in this study can be performed on a laptop (20 GB RAM, Quad core Intel I7). All relevant code and documentation can be found here: https://github.com/ruggleslab/phosphodisco.

### Input Data and Pre-processing

PhosphoDisco functions are built around the ProteomicsData class in Python, which requires phosphopeptide and protein input tables, assumed to be in the form of log_2_(relative abundance). Input data should be structured as samples as columns and proteins/phosphopeptides as rows (22). Detailed examples of both file structures can be found in supplemental Tables S2 and S3. We suggest that both tables be normalized for sample loading (*e.g.*, with median or upper quartile normalization). A function that can perform different normalization procedures is included (*column_normalize*) (supplemental Fig. S1). We do not recommend any row filtering and discourage row-normalizations like z-scoring, as maintaining different standard deviations between phosphosites is important for filtering before defining modules.

### Protein Normalization of Phosphopeptides

Phosphopeptide relative abundance data can be difficult to interpret because differences in abundance can be due to changes either in parent protein abundance or differences in phosphorylation state. To account for these confounding factors, PhosphoDisco includes a *normalize_phospho_by_protein* method which accounts for variation in abundance of the parent protein, and extracts variation due to changes in phosphorylation. The main caveat of this approach is that it will over-correct for peptides that are auto-phosphorylated or auto-dephosphorylated on kinases and phosphatases, respectively. In these cases, protein abundance and phosphorylation are interconnected, and therefore normalizing by the former will cancel out the latter. For this reason, PhosphoDisco also identifies putative regulator sites on kinases and phosphatases which can identify these special cases (see section Association With Possible Regulators below).

To complete protein normalization for each phosphopeptide, we train a model using regularized linear regression, using cross-validation (CV) to choose the regularization parameter (*linear_regression.RidgeCV* from scikit-learn (23)). We train the model with phosphopeptide abundance as the target and its parent protein abundance as the feature. We then use this model to predict phosphopeptide abundance values based on the parent protein abundance and subtract that value from the phosphopeptide abundance (convert to residuals) (Fig. 1*A*), resulting in normalized phosphopeptide abundance (*ProteomicsData.normed_phospho*). During normalization, only peptides that share at least as many non-missing values as the CV fold are retained; this step acts as a missing values filter. Regularization is important in the case of low correlation between a phosphopeptide and its parent protein (*e.g.*, if a lack of variation in protein abundance across the cohort exists) (Fig. 1*A*). Regularization values and CV fold are changeable parameters, and by default, the regularization values 0.00032, 0.0016, 0.008, 0.04, 0.2, 1, 5, 25, 125, 625, and 3 CV folds are used.

When combining different datasets (*e.g.*, different cancer types or tumor and normal samples), we recommend first performing this normalization for each subgroup, so in the case of two cancer types A and B, and their matched normals, we would normalize A-normals, B-normals, A-tumors, and B-tumors independently. This helps ameliorate non-linear scale differences between datasets.

### Filtering and Module Discovery

Prior to module discovery, we suggest that users retain only rows with high variance and low fraction of missing values from the protein normalized phosphopeptide table. This helps to reduce the required memory needed to run the pipeline and our default filters out the lower 50% of variance rows, after filtering out rows with more than 25% of missing values. Both of these steps are automatically performed by the PhosphoDisco pipeline. If using more than one cohort (*e.g.*, multiple cancer types or tumor and normal samples) we recommend performing this filtering step within each subgroup separately. For example, in the case of two cancer types A and B, and their matched normals, we would apply these filters to the groups of A-normals, B-normals, A-tumors, B-tumors, A, B, as well as A and B combined (supplemental Fig. S3). We then keep the union of all phosphosites that pass our filters in any of the groups. In our analysis of a combined LSCC and LUAD phosphoproteomic dataset, we have observed that upwards of 90% of retained phosphosites in the A/B example would come from filtering A and B combined.

To assign modules, PhosphoDisco completes a pairwise correlation between each phosphosite using the *assign_modules* function. Given that there is no clear precedent for a clustering algorithm most suited for module discovery in proteomics and phosphoproteomics, one method is to run a multitude of clustering algorithms (23). Additionally, hyperparameters are adjustable options that a statistical algorithm exposes to the user, allowing them to modify how the algorithm operates. For clustering algorithms, it is often not obvious which configuration is best for addressing a given question for a given data set. The built-in hypercluster package (24) enables the execution of a combination of multiple clustering algorithms with a range of hyperparameter sets. Each of these hyperparameter sets then clusters the data into its own set of modules. Hypercluster offers a variety of different metrics for evaluating which module set to choose, including the adjusted Rand index (25), which can tell us which module set is the most inclusive, *i.e.*, the most similar to all other module sets.

For small datasets, the *assign_modules* function runs the default parameters of hypercluster.MultiAutoClusterer.fit to find modules. For large datasets we recommend finding optimized clusters using the hypercluster Snakemake (21) pipeline in a distributed manner, then using the final labels and evaluations to pick the best clustering method for module detection (Fig. 1*B*). In the examples below, we chose the best modules by finding the top parameter sets based on the highest adjusted Rand index as suggested to identify the most robust modules. An example table with modules can be found in supplemental Table S4. Once modules are defined, a score for each module is calculated with the *calculate_module_scores* method by taking the mean of all members of the module per sample. Next users can use the *impute_missing_values* function to impute values missing in the *normed_phospho* attribute of the ProteomicsData object. This can be useful for plotting purposes for data sets with few missing values but can lead to artifacts with larger missingness percentages. For this sklearn.impute is used with a KNNImputer by default.

**Fig. 2:**
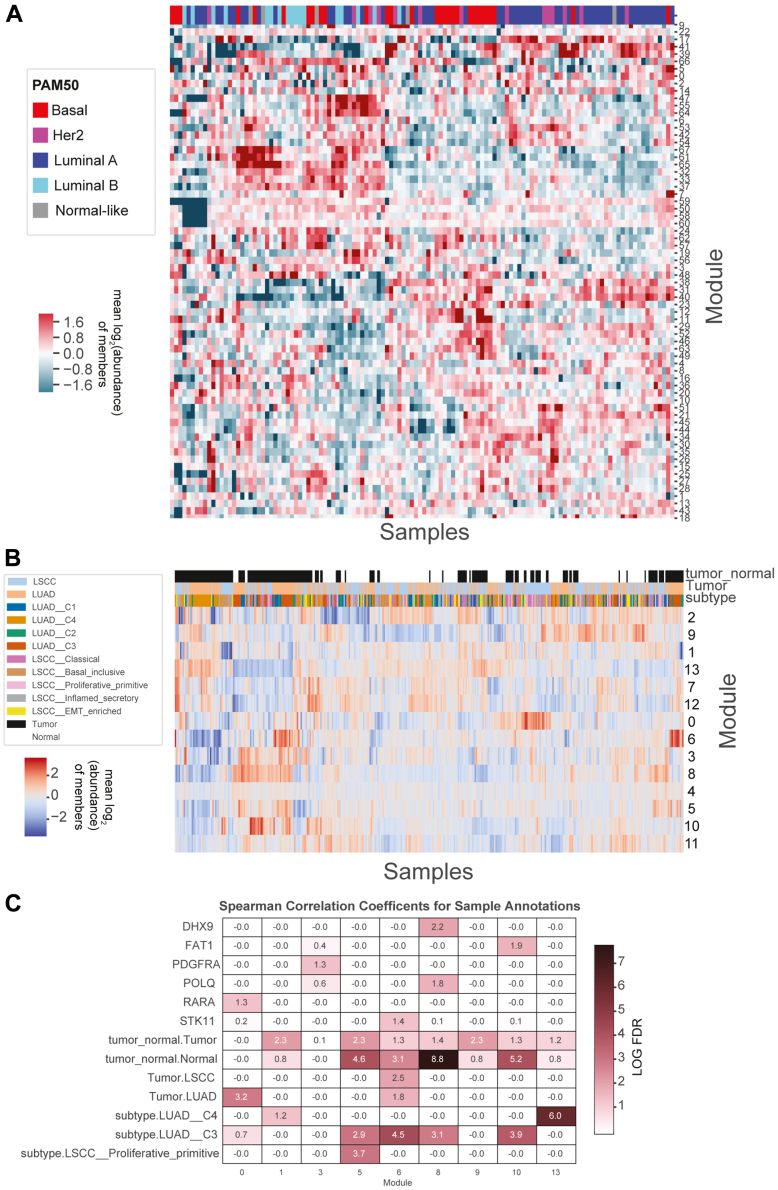
**Module selection and module scores of BRCA and combined LUAD-LSCC cancer datasets.** Module scores are calculated for each sample and each module by taking the log2 of the average abundance of all members in a module in a sample for (*A*) BRCA and (*B*) combined LUAD and LSCC cohorts. *C*, modules with significant correlations with sample annotations. DHX9, FAT1, PDGFRA, POLQ, RARA, STK11 indicate gene mutations.

PhosphoDisco includes a *visualize_modules* function that generates a clustered heatmap of protein-normalized phosphopeptides per sample for each co-regulated module (Fig. 2*B*). User-supplied sample annotations can be visualized alongside the heatmap to show relationships between module phosphosite levels and clinical features. This visualization is a key step for users to filter and interpret results. It is also important to visualize modules because clustering algorithms will often generate spurious modules, which can be manually removed by users from downstream analyses.

### Association With Clinical Variables

Users can also assess whether module scores are associated with user-supplied sample annotations such as tumor subtype or survival using the *calculate_annotation_association* method. These associations are useful for finding modules that may be relevant to diagnostic subgroups of tumors. An example of such an annotation table can be found in supplemental Table S5. An annotations DataFrame can be added to a ProteomicsData object using the *add_annotations* method. When *add_annotations* is applied, users must supply a list, defining which columns of the DataFrame are continuous or categorical variables, as categorical columns are split into binarized groups. Next, using the *calculate_annotation_association* function, users can calculate *p*-values for module enrichment per group per categorical variables, or correlation for continuous variables. The *p*-values are then corrected for multiple hypothesis testing (default is Benjamini Hochberg procedure (26)) from the statsmodels package (27) (Fig. 1*C*).

### Association With Possible Regulators

Proteins regulating the phosphorylation status of multiple phosphopeptides within a module are of particular interest as potential upstream targets for module inhibition and treatment. We therefore include methods to assist with nominating regulators for modules. Users can provide a list of gene names of putative regulators, matching identifiers in the top level of the index for the protein and phosphopeptide tables. The *collect_possible_regulators* function (supplemental Fig. S1 and supplemental Table S1) consolidates protein and phosphopeptide abundance data for all genes in that list. To prevent problems stemming from collinearity, this function also collapses features with higher than a user-set Pearson *R* value between them (default 0.95), by taking the mean values per sample. To prevent collapsing features, users can set the corr_threshold variable to a value above 1. After correlated features are collapsed, the remaining missing values are imputed with a sklearn.impute object (default is KNNImputer). The collected data is stored in the ProteomicsData.possible_regulators_data attribute. For this analysis, raw phosphopeptide data for each possible regulator is used, rather than protein-normalized data, to allow for the identification of auto-phosphorylation or auto-dephosphorylation, as discussed previously.

Once regulator data is collected, the *calculate_regulator_association* method finds the association between the vector of module scores and features in the possible_regulators_data attribute (Fig. 1*D*) for each pair of a regulator and a module. The method can be run in two modes: (a) it simply returns the correlation (default is Spearman), or (b) it builds a regularized linear model using possible_regulator_data as features and the modules scores at targets. There is also an option to transform the module scores so that the relationship being quantified between the regulators and modules scores is a sigmoid curve, as that is often the relationship between log(kinase abundance) and substrate concentration (28, 29) (supplemental Fig. S2*A*).

To aid in module prioritization, PhosphoDisco also provides the option to test the association of module scores with the abundances of a known set of kinase activation loop phosphosites (30) through the *correlate_kinase_activation_loop_phosphosites_with_module_scores* function. Kinase activation loop phosphorylation is strongly connected with kinase activity (30). Thus, this function can be used to further evidence for kinase activity beyond the more general *calculate_regulator_association* function. Users can also filter regulators by their known or predicted druggability using the druggable_regulator_heatmap function (31).

### Motif Analysis

Phosphatases and kinases often show preference for specific amino acid motifs, therefore analyzing the peptide sequences within a module can help further refine our list of potential regulators. Flanking amino acid sequences of each peptide can be defined if users provide an additional DataFrame with one column containing the corresponding protein identifier appropriate for the given fasta file (*e.g.*, the RefSeq isoform ID for that genome version) and one column with a comma separated ordinal value of the modified amino acid(s) along with a corresponding protein fasta file. Users can employ the *collect_aa_sequences* method (supplemental Fig. S1 and supplemental Table S6) to find the flanking amino acids for each modified site which will create the *module_sequences* attribute, containing dictionaries of amino acid sequences for each module.

The method *calculate_motif_enrichment* (supplemental Fig. S1 and supplemental Table S1) then finds amino acid motifs in clusters. The function aligns a user-set number of amino acids on either side of the modified site (default is 15, 7 on each side of the phosphorylation site). A *p*-value is assigned to the enrichment or depletion of each amino acid and is calculated using a Fisher’s exact test. Users can visualize raw counts or the enrichment log_10_(*p*-value) (Fig. 1*E*) of amino acids at each site. The function *visualize_aa_similarity* (supplemental Fig. S1) can then be used to detect multiple motifs in a single module. For each module, this function counts how many amino acids a peptide has in common with another peptide across a single module. It then visualizes these similarities in a clustered heatmap (Supplemental Fig. S2*B*). This allows users to identify multiple groups of similar peptides within a module.

### Gene Ontology Term and Post Translational Modification Set Enrichment

In addition to motif discovery, flanking amino acids can also be used to compare phosphorylation modules to known modified peptide sets from PTM-ssGSEA (32), which can help with module interpretation. Upon running *collect_aa_sequences* to collect sequences, the *ptm_ssgsea* function can be used to find enriched peptide sets. In addition, users can apply gene ontology enrichment tests on gene sets from each module, using gseapy and Enrichr (33, 34). Either of these results can be visualized as bar plots using the *visualize_set_enrichment* function (Fig. 1*F*, supplemental Fig. S1 and Supplemental Table S1). In addition, enrichment in protein complexes can be assessed using the same GO enrichment analysis method using protein complex databases (*e.g.*, NURSA_Human_Endogenous_Complexome) to specifically check for enrichment of protein complexes within modules.

### Sensitivity Analysis

The strength of the signal for a given module will depend, to some degree, on the number of samples. Therefore, to provide guidance on sample size and associated limitations, we completed a sample size sensitivity analysis based on input size. Specifically, we assessed PhosphoDisco’s ability to produce consistent results when applied to smaller cohorts by running the pipeline on 15 randomly-selected patient cohorts of size n = 25, n = 50, n = 75 or n = 100, resulting in 60 individual PhosphoDisco runs. This allowed us to determine if these smaller subsampled cohorts would consistently recapitulate our original findings. Using the breast cancer dataset and specifically module BRCA-63 as a test case, we determined the ability of PhosphoDisco to resolve the same phosphosites within each cluster at different cohort sizes based the adjusted rand index and percent of phosphosite overlap in BRCA-63 across the 15 runs. Patient samples were subsampled from the full CPTAC BRCA tumor dataset (n = 122) using the ShuffleSplit module from the python scikit-learn package to ensure phosphosite variability was as evenly distributed as possible.

## Results

### Discovery and Exploration of Putatively Co-regulated Phosphorylation Modules

To demonstrate the utility of PhosphoDisco to analyze proteomic and phosphoproteomic data, we provide two case studies, the first from a breast cancer cohort (BRCA, N = 122) (17) and the second integrating a lung adenocarcinoma (LUAD, N = 98) (16) and a lung squamous cell carcinoma (LSCC, N = 99) (15) cohort, all from the Clinical Proteomic Tumor Analysis Consortium (CPTAC). In addition to the tumor samples, both lung cohorts have matched normal samples from tumor-adjacent healthy lung tissue (N = 98 and N = 99 for LUAD and LSCC, respectively, Table 1).

### Breast Cancer Cohort Analysis

For our BRCA analysis, following protein normalization, we filtered out sites that had more than 25% missing values and took the top 50% of sites with the highest standard deviation. We then calculated the pairwise correlation between all sites and applied hypercluster to test several clustering techniques and hyperparameter combinations to find clusters of similar phosphosites (putatively co-regulated modules). To identify the most reproducible modules across hyperparameter settings, we determined how similar each set of labels is to each other, as measured by the adjusted rand index, which is a measure of the similarity between sets of labels (supplemental Fig. S4*A*). There were several sets of labels from the hdbscan algorithm (35) that were highly similar to almost all other sets of labels, due to hdbscan’s ability to dispose of sites that do not cluster well with anything. To keep the maximum number of phosphosites for downstream analysis, from this set of reproducible labels we chose the hyperparameters that led to the most labeled phosphosites. These criteria led to the selection of labels calculated by the hdbscan algorithm with a minimum cluster size of 4; resulting in 69 modules representing 1017 phosphosites (Fig. 2*A*, Table 1 and Supplemental Table S4).

In the clinic, the treatment strategy for breast cancer is determined by the presence or absence of three receptors: the estrogen receptor (ER), progesterone receptor (PR), and the human epidermal growth factor receptor (HER2). Patients with tumors presenting these receptors can receive therapy that prevents receptor signaling and downstream proliferation. Tumors lacking all three receptors (*i.e.*, triple-negative breast cancer, TNBC) have the worst prognosis and the most aggressive suggested treatment (36, 37, 38). Based on PAM50 subtyping (39, 40, 41, 42) there are four major molecular subtypes: Luminal A (LumA), Luminal B (LumB), Her2-enriched (Her2e), and Basal-like (Basal) with Basal tumors strongly enriched for TNBC. To test the utility of PhosphoDisco in breast cancer discovery, we focused on modules associated with the Basal tumor type.

### Module BRCA-63: A Basal Subtype-Enriched Cell Cycle Checkpoint Module

**Fig. 3:**
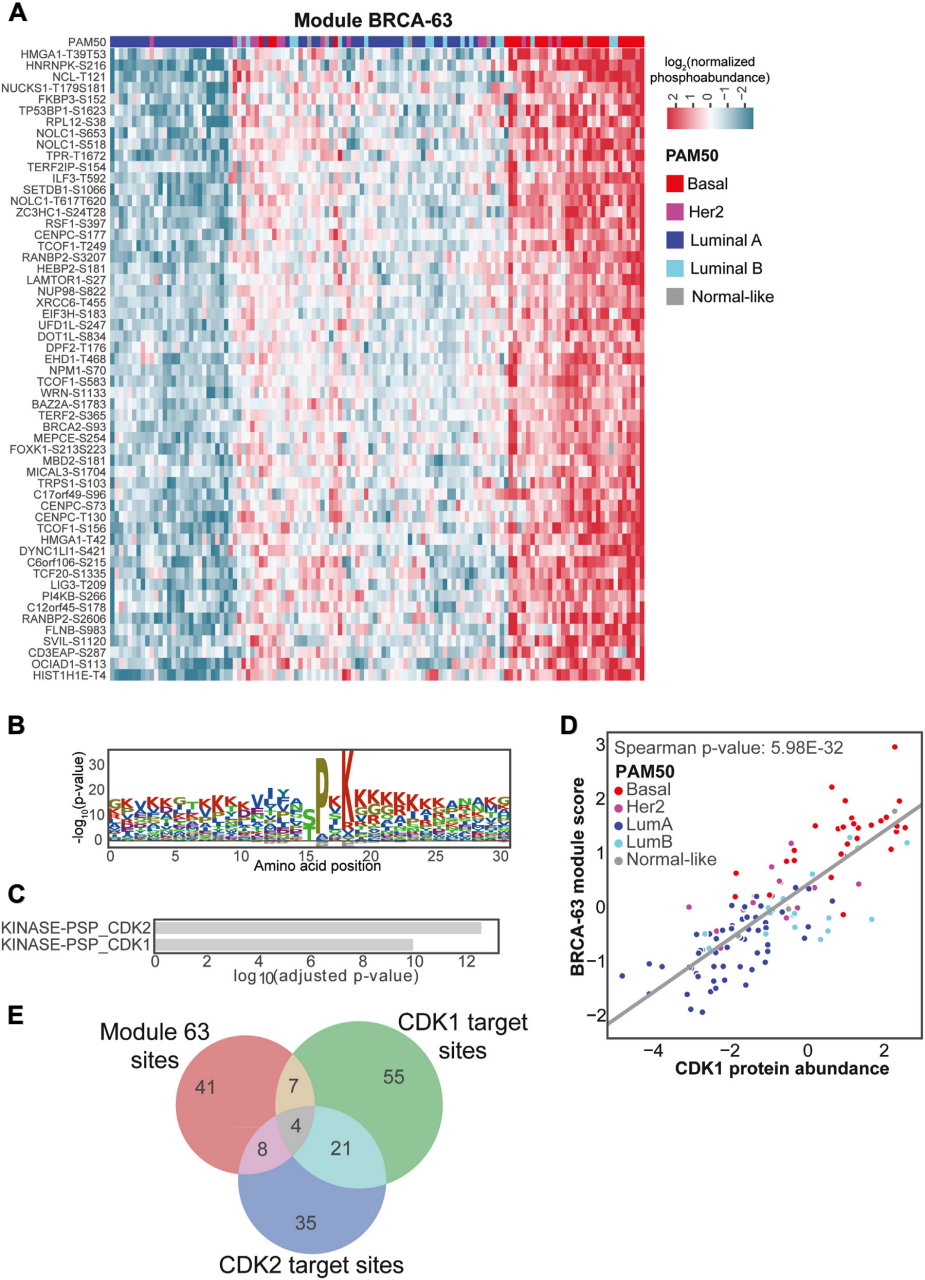
**Module BRCA-63.***A*, heatmap of normalized phosphosite abundance. *B*, module peptide motif. *C*, PTM SEA enrichment. *D*, overlap between kinase substrate phosphosite sets and module phosphosites. *E*, correlation between CDK1 protein abundance and module scores.

The majority of TNBCs are in the Basal subtype (50–75%), and the majority of Basal tumors are TNBC (>90%). Target discovery is especially important for patients with Basal subtype tumors, as they usually have poor prognosis and dearth of viable targets. As such, we first set out to prioritize modules that may represent key biological pathways active in this highest risk subtypes. To do this, we calculated an adjusted *p*-value representing the association of each PAM50 group with each module (43) identifying module BRCA-63 as having the strongest association with the Basal subtype (adjusted *p*-value = 1.8e-7) (Fig. 3*A*).

Module BRCA-63 appeared to be a cell cycle checkpoint signaling module, as it has an SP/TP motif, characteristic of CDK1 and CDK2 targets (Fig. 3*B*). Furthermore, the phosphosites in this module significantly overlapped with known CDK1 and CDK2 targets (adj *p* = 1.27e-8, 7e-10, respectively) (32, 44) (Fig. 3*C*), though the majority of the sites were not contained in the CDK1 or CDK2 substrate lists from PhosphoSitePlus (Fig. 3*E*). When looking for regulators of the module, we identified a strong correlation between the module score and CDK1 abundance (Fig. 3*D*), as well as other mitotic kinases such as TTK, AURKB and MASTL (Fig. 1*D*). Moreover, this module contains compelling novel targets of CDK1 and/or CDK2, such as serine 183 on EIF3H, a site whose phosphorylation has been shown to be functionally necessary for oncogenic proliferation (45), potentially pointing to a connection between cell cycle checkpoint activation and translational initiation control.

Interestingly, the putative cell cycle checkpoint module (BRCA-63) is separate from a module containing several KI67 phosphosites (module BRCA-48) (supplemental Fig. S5), which is a marker of proliferation and is broadly used as a prognostic test in breast cancer (46, 47, 48, 49). While both modules have high scores in Basal samples (Fig. 3*A* and supplemental Fig. S5*A*), the cell cycle checkpoint module is secondarily high in Her2e samples, while the proliferation module is also high in LumB samples (supplemental Fig. S5, *B* and *C*). This discrepancy indicates that proliferation and cell cycle checkpoints are separable pathways at the phosphorylation signaling level. The cell cycle checkpoint module is of particular interest, because CDK4/6 inhibitors (CDK4/6i) are currently being used to treat ER+ breast cancer (50), but there is a dearth of reliable biomarkers for the efficacy of this treatment (51, 52). Since CDK2 overactivation can compensate for CDK4/6i by phosphorylating the tumor suppressor Rb (53), the phosphorylation of peptides in this module could be excellent candidates as putative biomarkers for CDK4/6i resistance. The use of these biomarkers (along with others, such as measuring Rb loss) could expand the use of CDK4/6i to new patient cohorts.

### Down-Sampling to Assess Module Identification Sensitivity in Smaller Cohorts

To assess PhosphoDisco’s ability to produce consistent modules with smaller sample size inputs, we ran 15 randomly selected patient cohorts at four different cohort sizes (n = 25, n = 50, n = 75 or n = 100), resulting in 60 total runs. We then compared the modules identified by this subsampling to determine if smaller patient cohorts were able recapitulate the same modules that we found in our 122-patient cohort set, with a particular focus on BRCA-63 as a test case. Using the HBDSCAN-min-4 clustering method, the same method applied in our original analysis, we found that PhosphoDisco performed exceptionally well regardless of sample size. Comparing phosphosite overlap for module BRCA-63 and modules identified in our subsampled analysis, we found an average phosphosite overlap of 86.65%, 96.20%, 97.99%, and 98.44% for n = 25, 50, 75, and 100 patient cohorts, respectively (supplemental Fig. S6*A*) between the original BRCA-63 module and its most similar cluster in each of the runs.

Although the high performance for the n = 25 patient cohorts suggests that we can confidently identify modules from cohorts of 25 patients or more, we did note higher module variability as sample size decreased. Using an adjusted rand index for which BRCA-63 phosphosites were clustered across the 15 runs at each cohort size level we found that n = 25 had the lowest adjusted rand index at 0.06. This jumps considerably to 0.80 for n = 50 patients and is a perfect score of 1.0 for n = 75 and n = 100 patients (supplemental Fig. S6*B*). This suggests that n = 25 patients may be sufficient to find the majority of the important phosphosites for key modules within your data, however additional patients can substantially increase consistency across runs. We suggest that, when feasible, users use a similar bootstrapping method to test the robustness of their modules based on a random down sampling approach.

### Non-Small Cell Lung Cancer Cohort Analysis

To demonstrate PhosphoDisco’s functionality in the analysis of combined datasets, we combined tumor and normal samples from a lung squamous cell carcinoma (LSCC) (15) and lung adenocarcinoma (LUAD) datasets (16). Non-small cell lung cancer (NSCLC) accounts for around 80% of lung cancers (54). The two most common NSCLC subtypes are LUAD (50%) and LSCC (40%) (54). Subtyping within LSCC and LUAD is not as clearly established as it is in breast cancer.

Phosphoproteomics data from LSCC tumors, LSCC normals, LUAD tumors, and LUAD normals were normalized separately and combined following the procedure outlined in supplemental Fig. S3. For the combined data set, we retained phosphopeptides that passed our filters (maximum 25% missing values, top 50% of highest standard deviation) when looking at the samples in any one of several different combinations: (i) LSCC tumors, LSCC normals, LUAD tumors, and LUAD normals separately; (ii) all LSCC samples and all LUAD samples separately; and, (iii) all lung samples together. We then took the pairwise correlation between all sites. To find clusters of similar phosphosites (putatively co-regulated modules), we applied hypercluster to test several clustering techniques and hyperparameter combinations.

To find the most reproducible modules across hyperparameter settings, we determined how similar each set of labels are to each other, as measured by the adjusted rand index (supplemental Fig. S4*B*). As seen previously (supplemental Fig. S4*A*), there were several sets of labels from the hdbscan algorithm (35) that were highly similar to almost all other sets of labels and again chose hyperparameters that led to the most labeled phosphosites. These criteria led to the selection of labels calculated by the hdbscan algorithm with a minimum cluster size of 9; these labels contained 14 modules representing 1684 phosphosites (Fig. 2*B* and supplemental Table S7). Nine of these clusters were found to be significantly correlated with a clinically relevant feature including mutation status and cancer subtype (Fig. 2*C*).

### Module Lung-3: Phosphorylated PRKC Isozymes Enriched Module in Lung Tumors

**Fig. 4:**
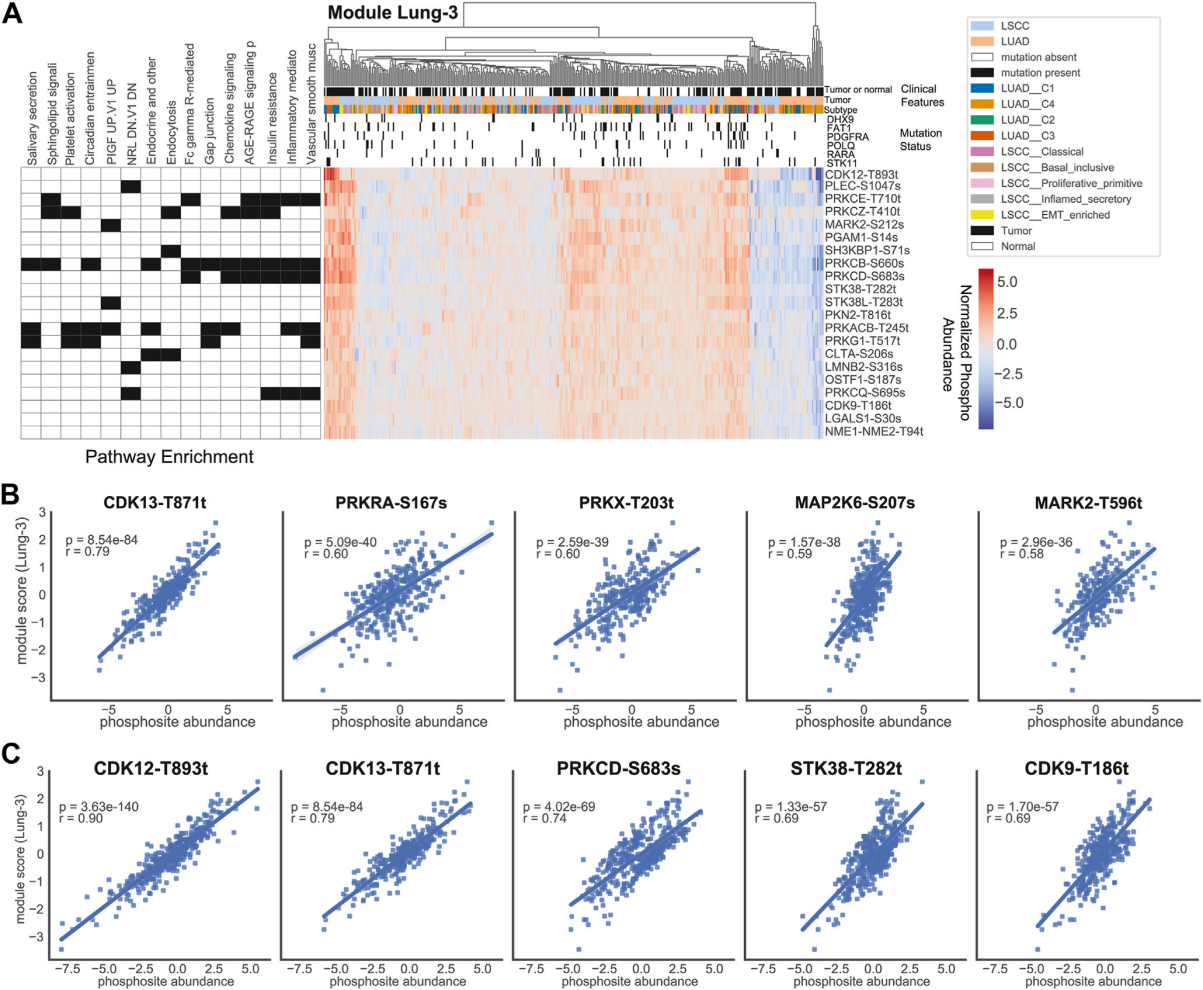
**Module Lung-3.***A*, module plot for Lung-3, including heatmap of normalized phosphosites for samples *versus* module phosphosites, GO-term annotations, and annotations. *B*, top nominated regulators that are not also part of the module phosphosite list. *C*, top nominated regulators regardless of module membership. Trendlines in (*B*) and (*C*) are not representative of listed r and *p*-values, which were calculated using spearman correlation (scipy.stats.spearmanr with default arguments).

Expression of PRKC isoforms has been associated with poor prognosis in non-small cell lung cancer (NSCLC) (55). In our combined LSCC and LUAD dataset, we identified module Lung-3, which was made up of 21 phosphosites (Fig. 4*A*). These phosphosites included PRKC isoforms B, D, E, Q, all of which have been associated with reduced drug sensitivity, increased cell survival, proliferation, invasion, migration, evasion of apoptosis, anchorage-independent growth, progression, chemotaxis, as well as cell cycle progression (55) making this module potentially interesting for therapy. We found phosphosites of both CDK12 and CDK13 to be strongly correlated to the module scores of this module (CDK13-T871: *p* < 9e-84, corr = 0.79; CDK12-T893: *p* < 4e-140, corr = 0.90) (Fig. 4, *B* and *C*), where the CDK12 site is part of the module, and the CDK13 site is not. Additional potential regulators included PDGFA, PRKCD, PRKCZ, and PRKRA, indicating a potential role for ceramide metabolism (56) in this module (Fig. 4, *B* and *C*). Further, we found that the Lung-3 module scores were significantly correlated with a mutation in the platelet-derived growth factor receptor alpha gene PDGFRA (adj-*p* = 0.036) (Fig. 2*C*). This gene has been shown to play a role in wound healing and tumor progression in a variety of cancers (55). In summary, this module encompasses multiple co-correlated paralogs of PRKC that may be co-regulated by CDK12/CDK13, potentially *via* the ceramide pathway and PRKRA. Many of the protein kinase C phosphopeptides in this module are also highly similar to each other while not being identical (supplemental Fig. S7*B*), which would be consistent with these phosphosites being phosphorylated by the same kinase or family of kinases. Interestingly, we found a highly similar module (*p* < 2e-10) in our breast cohort (BRCA-38), containing phosphosites in PRKCB and PRKCD as well as CDK12 and CDK13 (supplemental Fig. S7, *C* and *D*).

### Module Lung-5: Cell Cycle/Proliferation Module

**Fig. 5:**
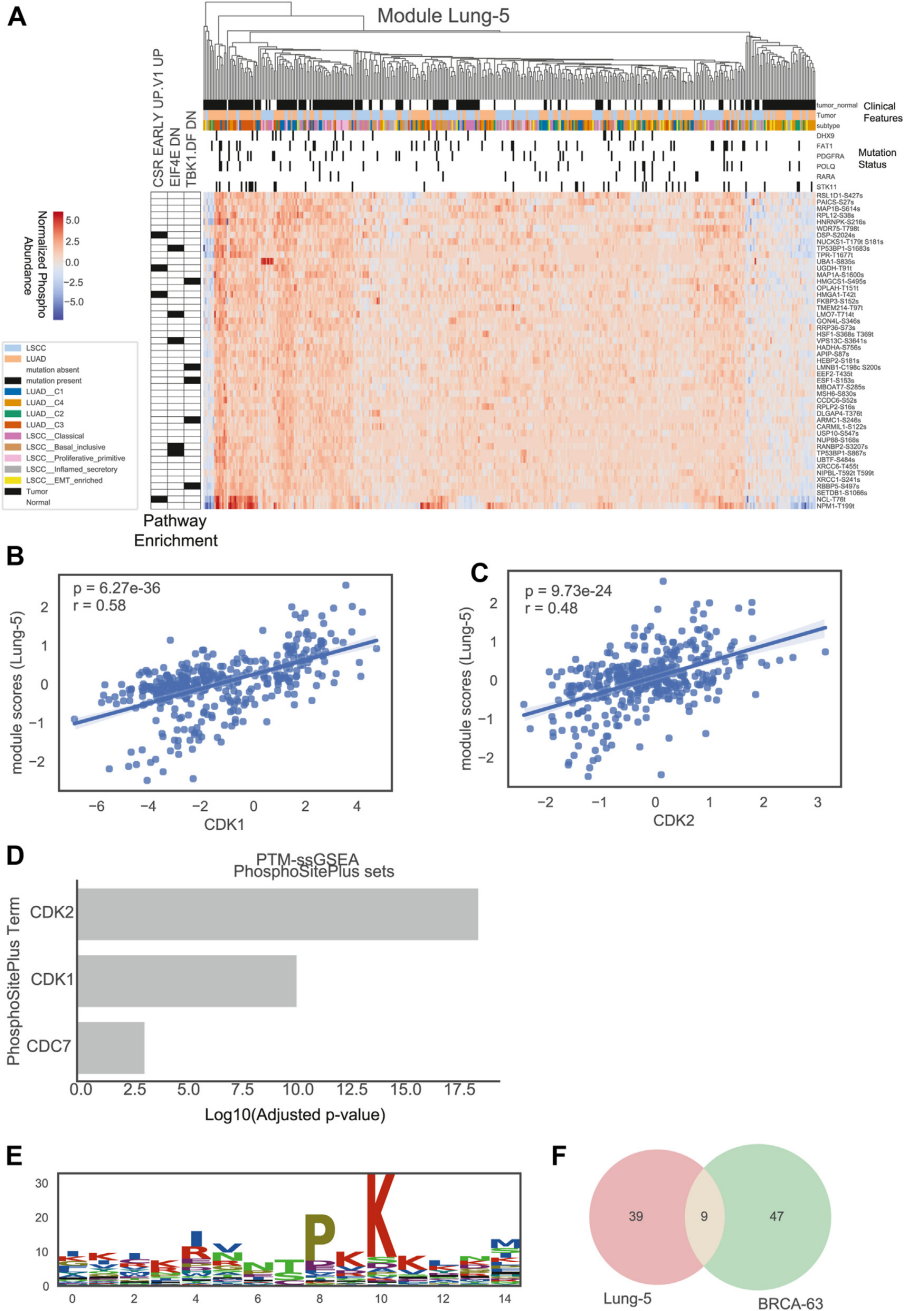
**Module Lung-5.***A*, module plot for Lung-5, including heatmap of normalized phosphosites for samples *versus* module phosphosites, GO-term annotations, and annotations. Correlation between module scores of Lung-5 and (*B*) CDK1 and (*C*) CDK2 expression. *D*, PTM ssGSEA enrichment analysis of module 5 against PhosphoSitePlus sets. *E*, Lung-5 peptide motif of 15 length peptides centered on module phosphosites. *F*, overlap between phosphosites in Lung-5 and BRCA-63.

An additional module, Lung-5, was identified as being associated with the proliferative primitive subtype of LSCC, as well as the LUAD proximal proliferative subtype C3 (*p*-values < 0.0002) (16) (Fig. 5*A*). Lung-5 module scores were found to have high correlation with CDK1 and CDK2 expression (Fig. 5, *B* and *C*) and were found to contain a significant number of known CDK1 and CDK2 phosphosites, as determined by PTM-SEA (Fig. 5*D*). Further evidence for CDK1 and CDK2 as regulators for this module, is the S/T-P-x-K peptide motif enriched in the phosphosites of this module (Fig. 5*E*), which is consistent with the known CDK1 motif (57). The site TPR-T1677 in module Lung-5 was previously observed in cell lines (58) as a minorly abundant phosphosite with unknown regulator. The same study (58) found TPR-S2059 to be phosphorylated by CDK1 in cell lines which we did not observe in our data set. This suggests that TPR-T1677 may also be phosphorylated directly by CDK1 and may in fact be the major phosphosite.

Further, proteins in this module were found to be significantly enriched (FDR < 0.05) in five protein complexes (BL840, AB81, BL373, BL6690, SC-126) based on the NURSA protein complexes database, a dataset made up of protein-protein complex associations that were identified by protein in complex recovered using Immunoprecipitation-MS (59). RANBP2, DSP, NPM1, XRCC6, TP53BP1, RPLP2 and HNRNPK were found in all five complexes identified. Interestingly, the majority of these genes are known binders of the tumor suppressor, TP53. This is consistent the associations found with CDK1/2 as p53 is known a downstream kinase substrate for these kinases.

Interestingly, this module has substantial overlap (*p* < 6e-14) with the breast cancer module BRCA-63 (Figs. 3 and 5*F*, supplemental Table S8). Three of the parent proteins of the phosphosites these modules have in common (HMGA1, HNRNPK, XRCC6) are known binding proteins of HMGB1, an important regulator of homeostasis in airway epithelial cells, as well as regulating immune and inflammatory responses (60).

## Discussion

### Zeroing in on Biomarkers and Novel Targets

While certain signaling pathways are well-established targets for cancer treatment, determining relevant players in a particular disease is challenging. We show that PhosphoDisco can help users identify and interpret co-regulated signaling modules in phosphoproteomic datasets. Applying PhosphoDisco to a breast cancer data set, we identified a putative cell cycle checkpoint module, which nominated novel targets of cell cycle kinases, drawing a potential link between cell cycle checkpoints and translation initiation control. In addition, the members of this module are good candidates to be tested as biomarkers of resistance to CDK4/6 inhibition. Lastly, PhosphoDisco was able to identify modules associated with mutation status and other relevant clinical features in lung cancer (Fig. 2*C*) further highlighting its ability to assists in processing and prioritizing phosphoproteomics data to generate hypotheses about biomarkers and novel therapeutic targets.

### Pan-Cancer Phosphorylation Signaling Pathways

Results from a preliminary pan-cancer PhosphoDisco analysis hint that phosphorylation modules found across cancers are more similar to each other than to current databases of kinase-substrate sets. The reproducibility of some of these modules increases the confidence in the approach taken by PhosphoDisco. The signaling pathways repeatedly found in CPTAC phosphoproteomics data could redefine key phosphorylation cascades, narrowing down the scope of downstream validation experiments across cancer biology. In particular, PhosphoDisco can be used for the design of combination kinase inhibitor therapy, where it can identify kinases likely to be in the same or different pathways. This can guide experiments testing whether inhibition of two kinases would be additive or redundant, or simply be effective in different populations (61, 62, 63). Additionally, PhosphoDisco could be used to find redundant kinases in the same pathway, for which only simultaneous inhibition would lead to an effect.

### Limitations

Since individual phosphosites are typically only quantified based on the measurement of one peptide, we expect variation in measurements to be larger for phosphosite level analysis compared with protein quantification, where often several peptides can be used. Further, phosphorylation data is very high in its level of missingness. These inherent limitations require tools specific to this data structure, and PhosphoDisco was designed to support both the larger quantitative error rate and the missingness in phosphorylation data. The sparsity of this data type also complicates our ability to comprehensively assess ‘gold standard’ kinase-substrate sets in our data but we expect that this will improve as mass spectrometry-based proteomics technology continues to advance.

### Expanded Use Cases

Each function provided by PhosphoDisco could be useful as steps in other custom analysis pipelines. We particularly anticipate the protein normalized phosphopeptide abundance to be of broad use for other analyses, where determining whether differences in abundance are due to phosphorylation or parent protein expression changes. While this tool is currently most suited to data sets from CPTAC and similar consortia, there are increasing numbers of research programs that are using phospho-enriched shotgun mass spectrometry to study different systems (64). PhosphoDisco could potentially be used on any analogous PTM-enriched relative abundance mass spectrometry data, such as acetylproteomics. Acetylation-based signaling is chronically understudied, making high throughput experiments more difficult to interpret, though it is evidence that acetylation signaling could be key to understanding breast cancer progression (65) and many other cancer types (66, 67).

## Data Availability

All data is available in public CPTAC repositories (https://gdc.cancer.gov/about-gdc/contributed-genomic-data-cancer-research/clinical-proteomic-tumor-analysis-consortium-cptac).

## Supplemental data

This article contains supplemental data.

## Conflict of interest

The authors declare that they have no known competing financial interests or personal relationships that could have appeared to influence the work reported in this paper.

## Supplemental Data

Supplemental Figure S1 **PhosphoDisco workflow overview.** Workflow diagram showcasing the use of PhosphoDisco functions and the PhosphoDisco pipeline in the usual PhosphoDisco workflow. Boxes starting with a $ sign indicate commands run on the commandline. Filled boxes indicate input files/DataFrames supplied by the user/ produced by PhosphoDisco. Filled boxes like .module_scores that start with a dot signify attributes being added to the ProteomicsData object.

Supplemental Figure S2 **Kinase activity and motif estimation.***A*, sigmoid relationship between kinase abundance and phosphorylation. *B*, commonalities between two peptides and commonality matrix.

Supplemental Figure S3 **LSCC and LUAD normalization and filtering scheme for phosphorylation data.** Samples are separated by tumor/normal and LSCC/LUAD categories and phosphosite levels are normalized by parent protein abundance. Samples are then combined into groups hierarchically according to categories (tumor/normal, LSCC/LUAD), and all sample groups (blocks with dashed outlines) are subjected to missingness filtering (25% missingness tolerated) and standard deviation filtering (top 50% kept). The resulting list of phosphosites is combined and deduplicated.

Supplemental Figure S4 Phosphopeptide clustering and adjusted Rand Score for (*A*) breast and (*B*) combined LSCC & LUAD datasets.

Supplemental Figure S5 **Module BRCA-48.***A*, heatmap of protein-normalized phosphorylation abundance of members of BRCA-48. *B*, scatter plot showing the relationship between BRCA-48 and BRCA-63. *C*, boxplot showing the distribution of module scores for BRCA-48 and BRCA-63 in PAM50 subgroups.

Supplemental Figure S6 **Sample size sensitivity analysis.***A*, phosphosite overlap compared to n=122 and *B*, adjusted rand index for module BRCA-63 in 15 randomly chosen sets of n = 25, n = 50, n = 75 and n = 100.

Supplemental Figure S7 **Module Lung-3.***A*, Lung-3 peptide motif of 15 length peptides centered on module phosphosites. *B*, peptide overlap for 15 length peptides centered on module phosphosites. Color indicates number of overlapping amino acids. *C*, Venn diagram of phosphosites of Lung-3 and BRCA-38. *D*, analogous module found in the breast cohort (BRCA-38).

Supplemental Table S1 Function descriptions for core PhosphoDisco functions. Listed are core PhosphoDisco functions, with requirements, outputs and a description. For further information use the official documentation at https://github.com/ruggleslab/phosphodisco.

Supplemental Table S2 Example phosphorylation input data file. The rows are phosphosites, described by the first two columns *gene_symbol*, and *variable_sites_names*. The remaining columns represent patient samples. This data file is also used in the PhosphoDisco tutorial notebook https://github.com/ruggleslab/phosphodisco.

Supplemental Table S3 Example protein input data file. The rows are proteins, described by the first column *gene_symbol*. The remaining columns represent patient samples. This data file is also used in the PhosphoDisco tutorial notebook https://github.com/ruggleslab/phosphodisco.

Supplemental Table S4 Breast cancer modules output file. Module information for phosphosites from breast cancer dataset.The rows are phosphosites, described by the first two columns *gene_symbol*, and *variable_sites_names*. The remaining column *HDBSCAN;min_cluster_size-4* assigns a module number to each of the phosphosites, according to the output of running HDBSCAN with the parameter min_cluster_size set to 4. A regular PhosphoDisco output module file would also include rows which are assigned −1 to signify that this phosphosite has not been assigned to a module. This data file is also used in the PhosphoDisco tutorial notebook, and the −1 rows have been removed, leaving only phosphosites which have been assigned a module.

Supplemental Table S5 Example sample annotation input data file. The rows are patient samples, and the columns sample annotations. This data file is also used in the PhosphoDisco tutorial notebook.

Supplemental Table S6 DataFrame format for motif analysis. Each row represents a phosphosite. The column *gene_symbol* is the gene symbol, *variable_sites_names* will be concatenated with *gene_symbol* for plotting purposes, *protein_id* is the ID to be used to fetch the protein entry which matches the phosphosite, from the supplied fasta file, *variable_sites* the numerical indices of phosphorylated amino acids in the protein, and lastly *module* which is the module the phosphosite is assigned to.

Supplemental Table S7 Lung cancer modules. Module information for phosphosites from combined LSCC and LUAD data set. Module −1 means no module was assigned.

Supplemental Table S8 Phosphosite Overlap of BRCA-63 with Lung-5 module. (A) All phosphosites found in both the modules BRCA-63 and Lung-5. (B) Phosphosites in found in module Lung-5 but not in BRCA-63. (C) Phosphosites found in module BRCA-63 but not in Lung-5.
