# Supplementary figures and images for: PhosphoDisco: A Toolkit for Co-regulated Phosphorylation Module Discovery in Phosphoproteomic Data
Source: Mol Cell Proteomics. 2023 Jun 30;22(8):100596. doi: 10.1016/j.mcpro.2023.100596 (PMC10416063; doi:10.1016/j.mcpro.2023.100596)

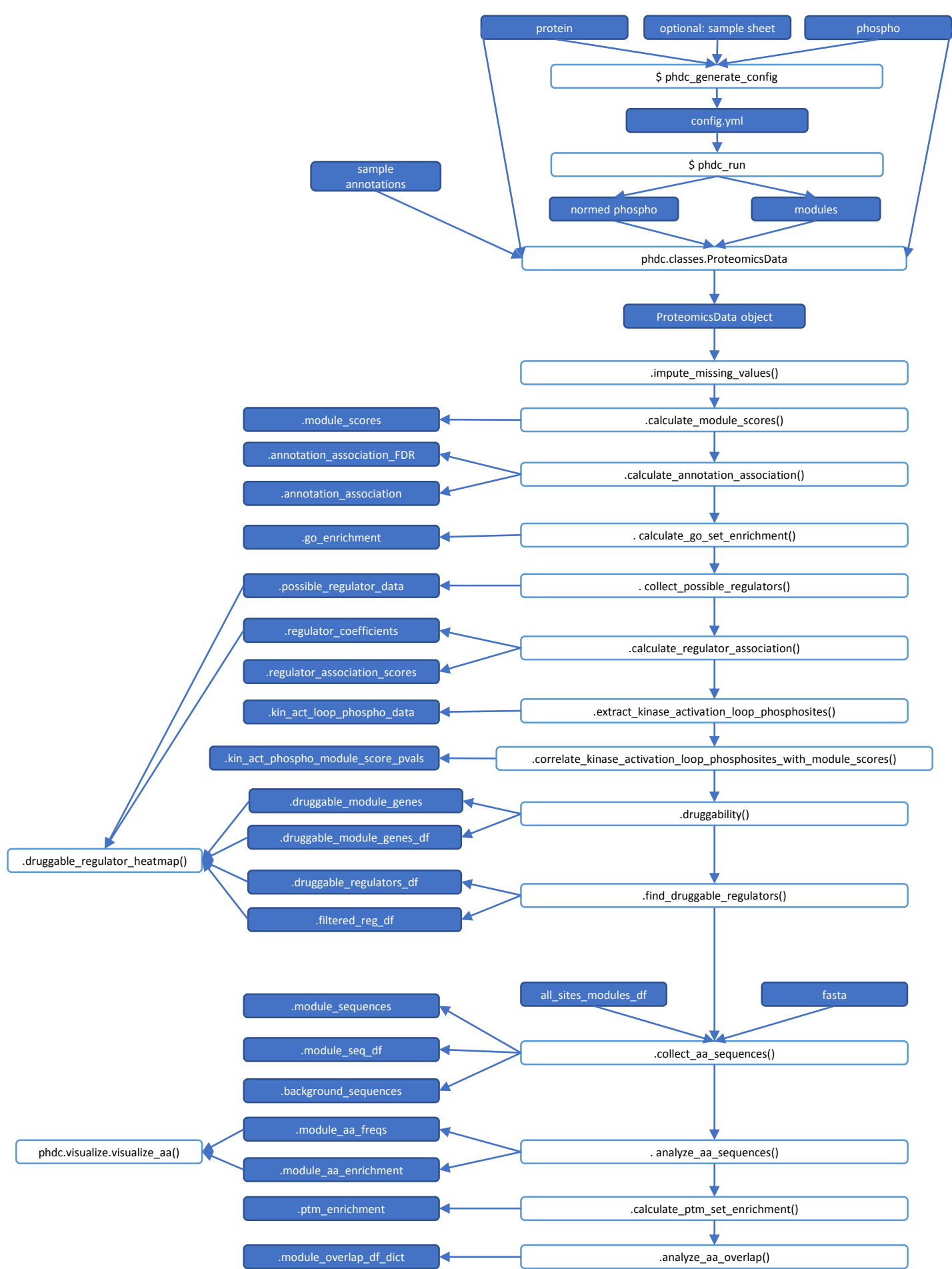

Supplement: Supplemental Figure S1 — PhosphoDisco workflow overview. Workflow diagram showcasing the use of PhosphoDisco functions and the PhosphoDisco pipeline in the usual PhosphoDisco workflow. Boxes starting with a $ sign indicate commands run on the commandline. Filled boxes indicate input files/DataFrames supplied by the user/ produced by PhosphoDisco. Filled boxes like .module_scores that start with a dot signify attributes being added to the ProteomicsData object. [file mmc1.pdf]

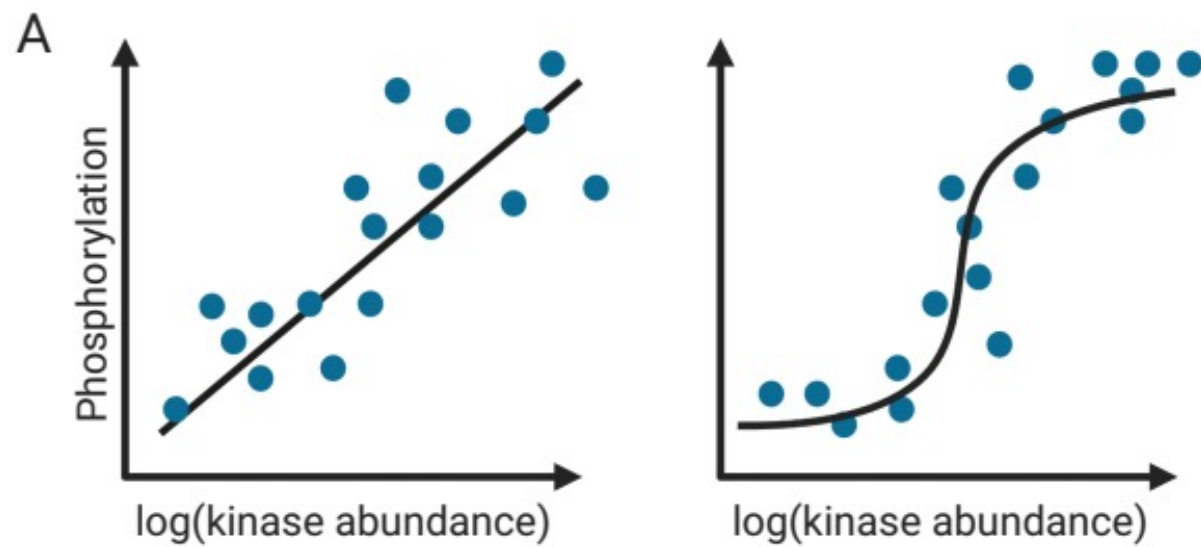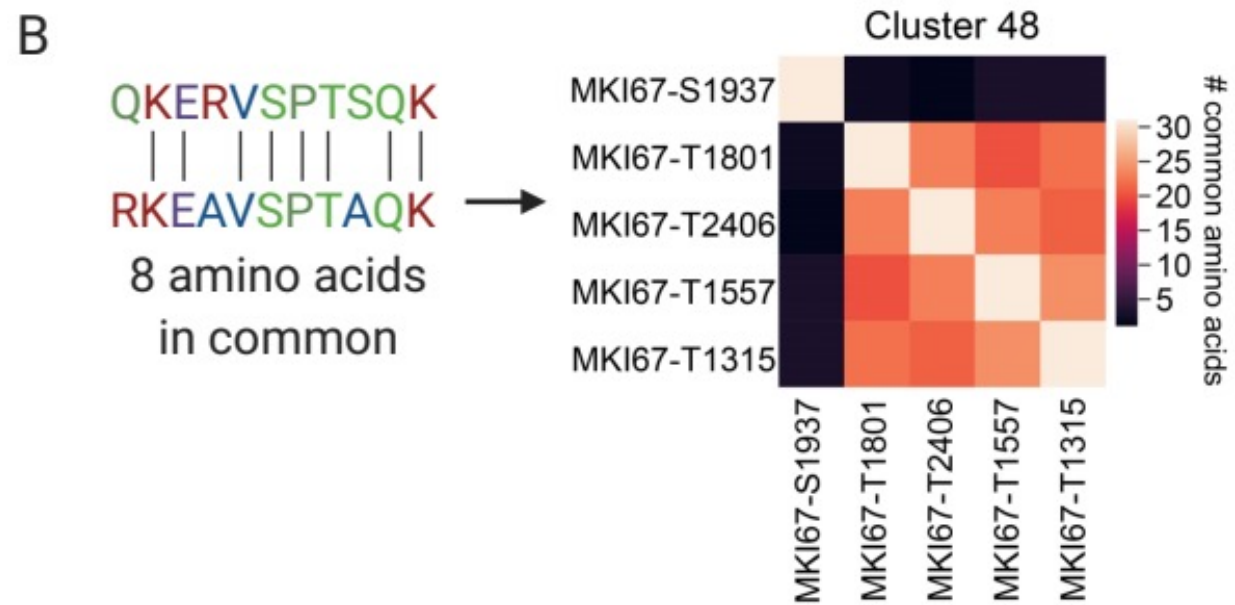

Supplement: Supplemental Figure S2 — Kinase activity and motif estimation.A, sigmoid relationship between kinase abundance and phosphorylation. B, commonalities between two peptides and commonality matrix. [file mmc2.pdf]

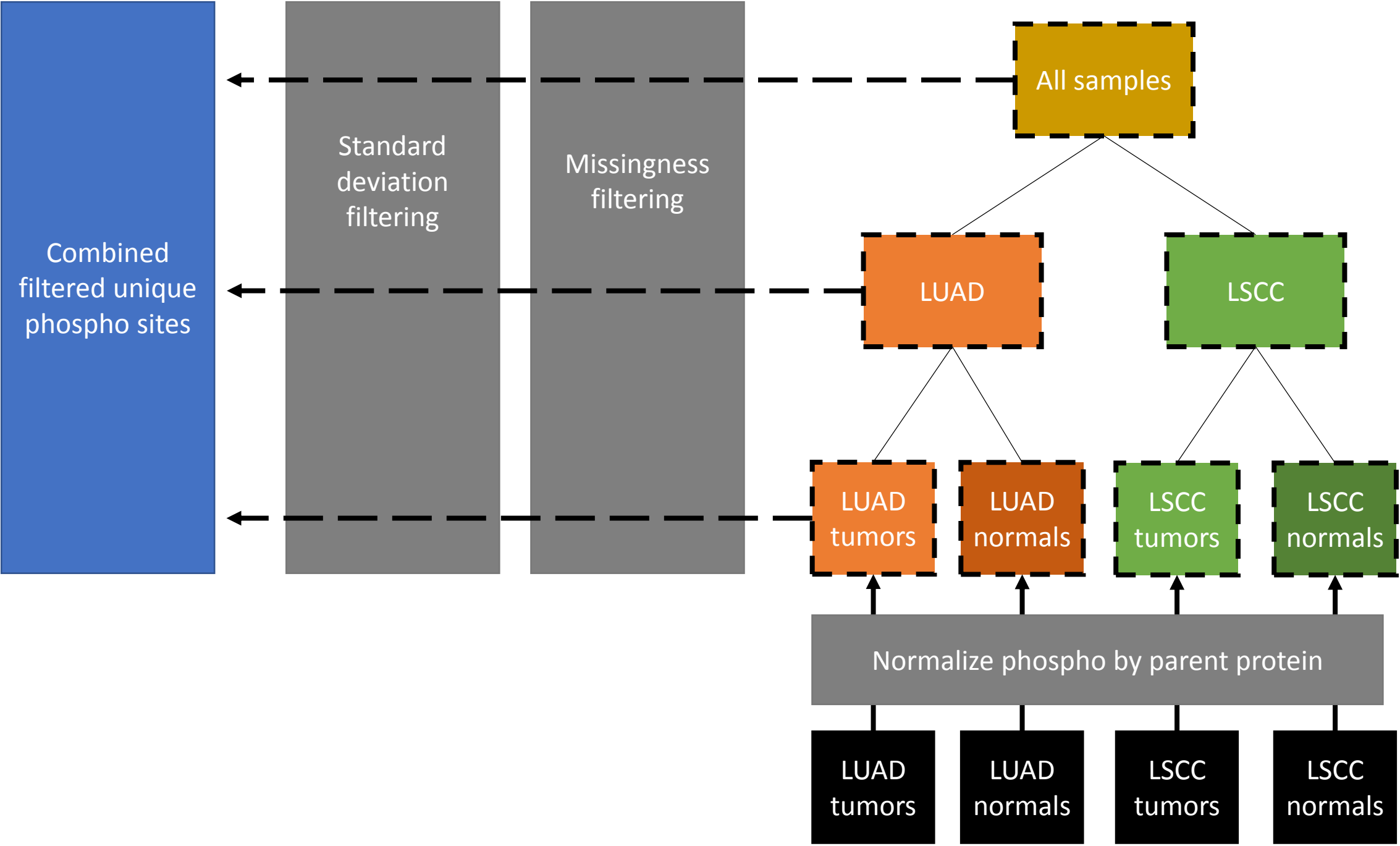

Supplement: Supplemental Figure S3 — LSCC and LUAD normalization and filtering scheme for phosphorylation data. Samples are separated by tumor/normal and LSCC/LUAD categories and phosphosite levels are normalized by parent protein abundance. Samples are then combined into groups hierarchically according to categories (tumor/normal, LSCC/LUAD), and all sample groups (blocks with dashed outlines) are subjected to missingness filtering (25% missingness tolerated) and standard deviation filtering (top 50% kept). The resulting list of phosphosites is combined and deduplicated. [file mmc3.pdf]

A

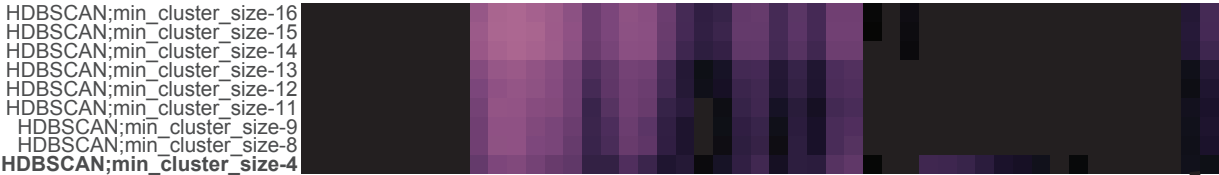

B

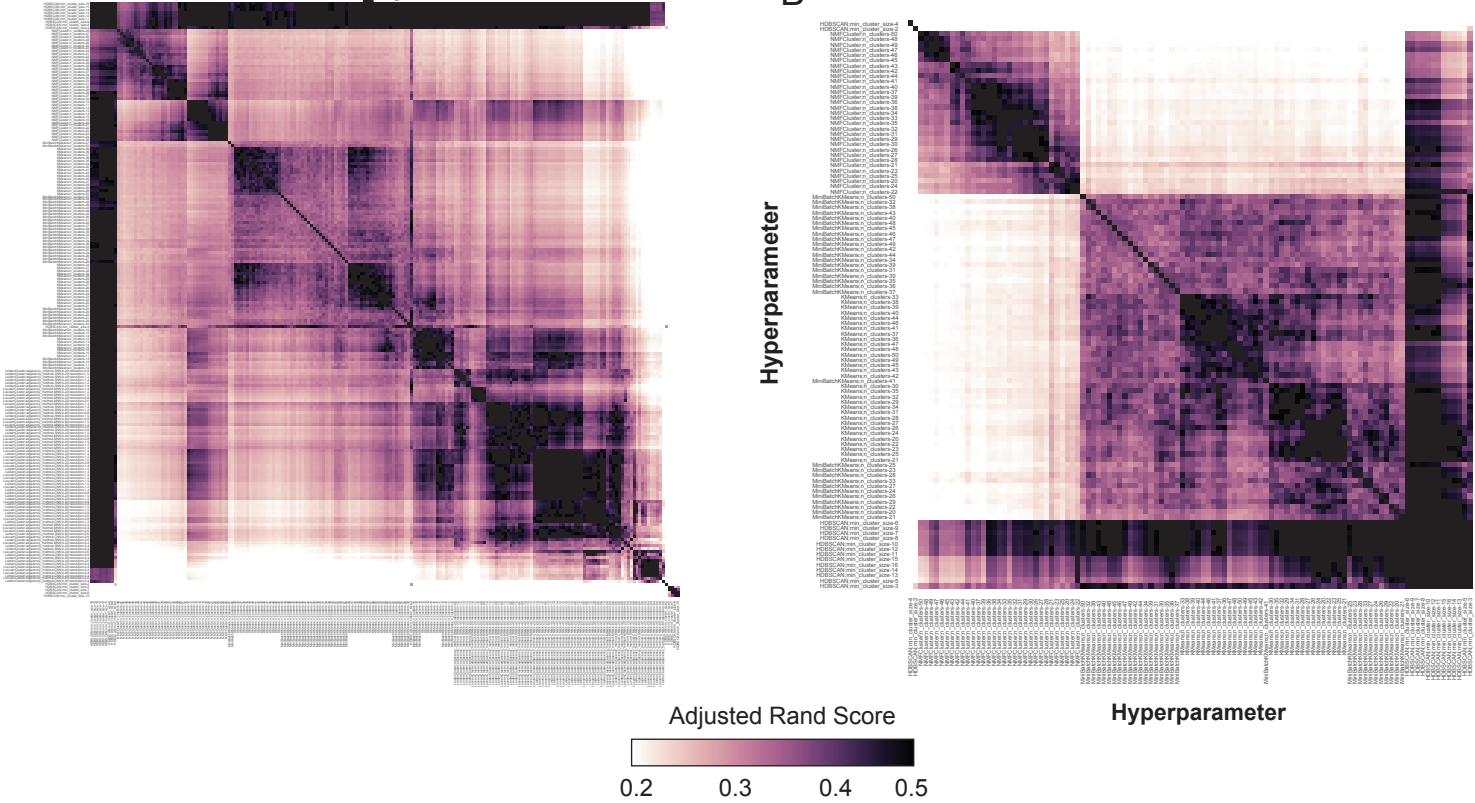

Supplement: Supplemental Figure S4 — Phosphopeptide clustering and adjusted Rand Score for (A) breast and (B) combined LSCC & LUAD datasets. [file mmc4.pdf]

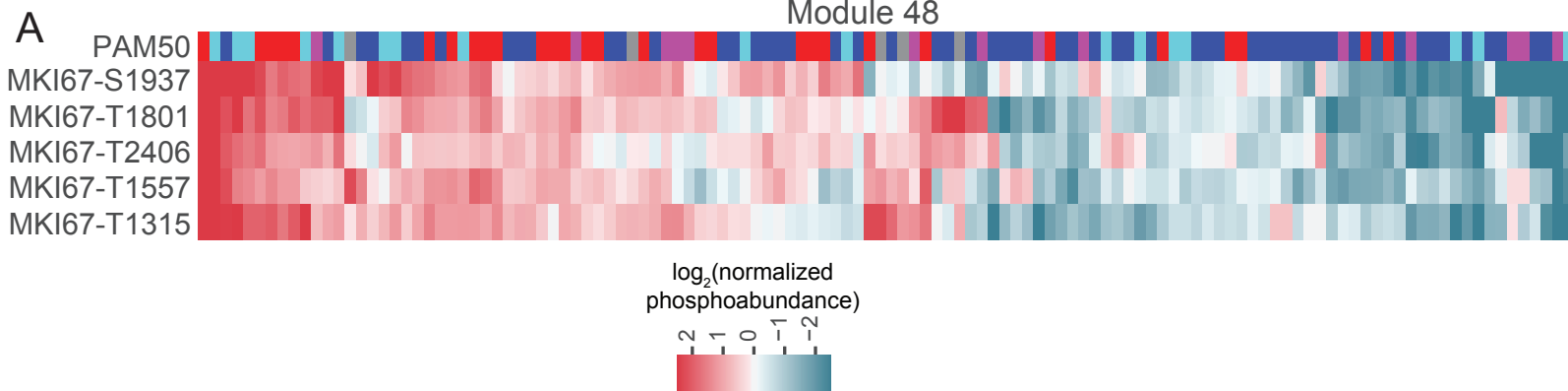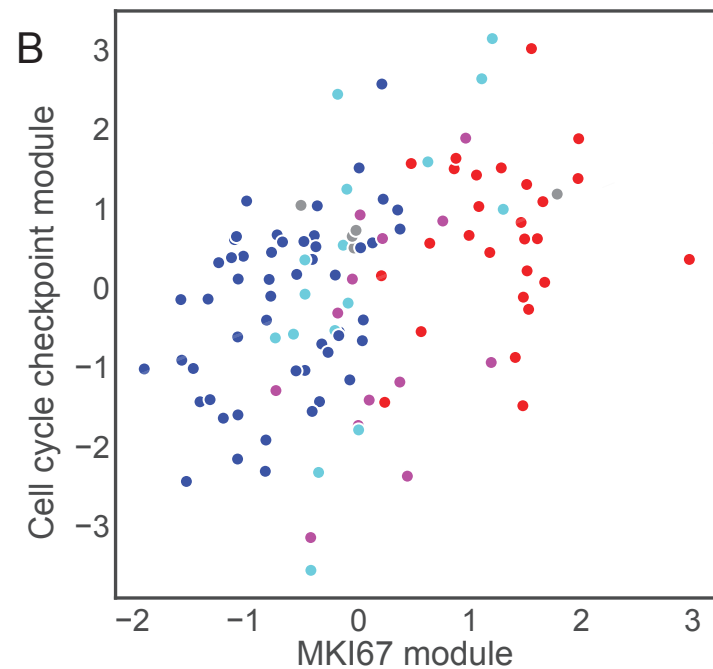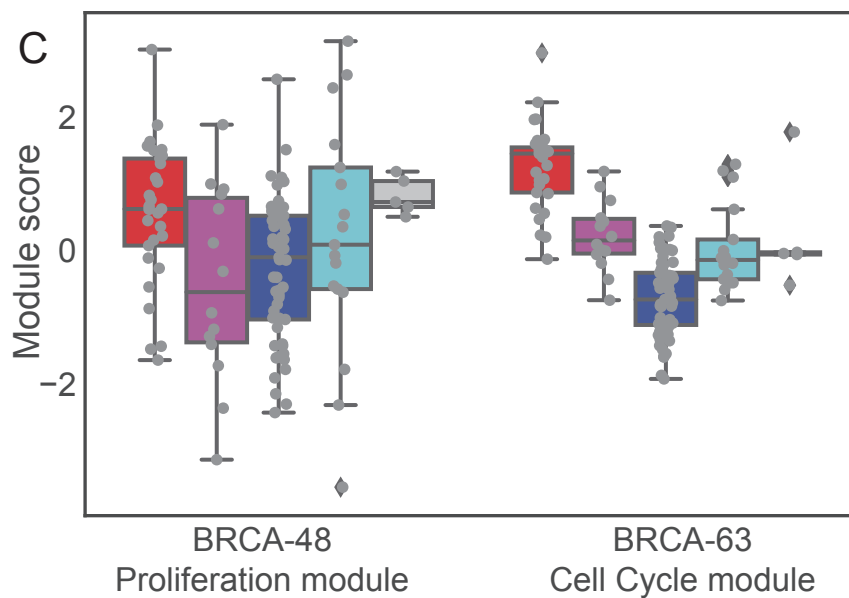

Supplement: Supplemental Figure S5 — Module BRCA-48.A, heatmap of protein-normalized phosphorylation abundance of members of BRCA-48. B, scatter plot showing the relationship between BRCA-48 and BRCA-63. C, boxplot showing the distribution of module scores for BRCA-48 and BRCA-63 in PAM50 subgroups. [file mmc5.pdf]

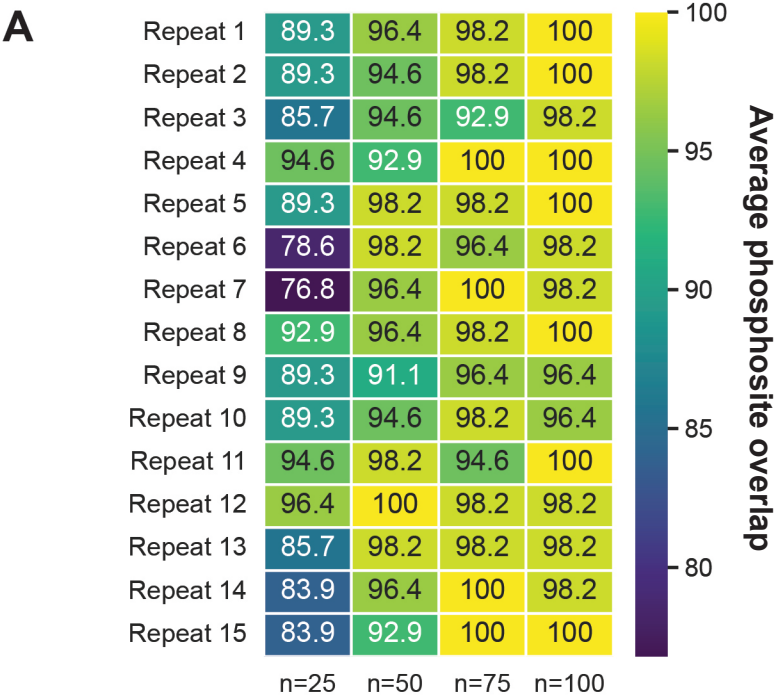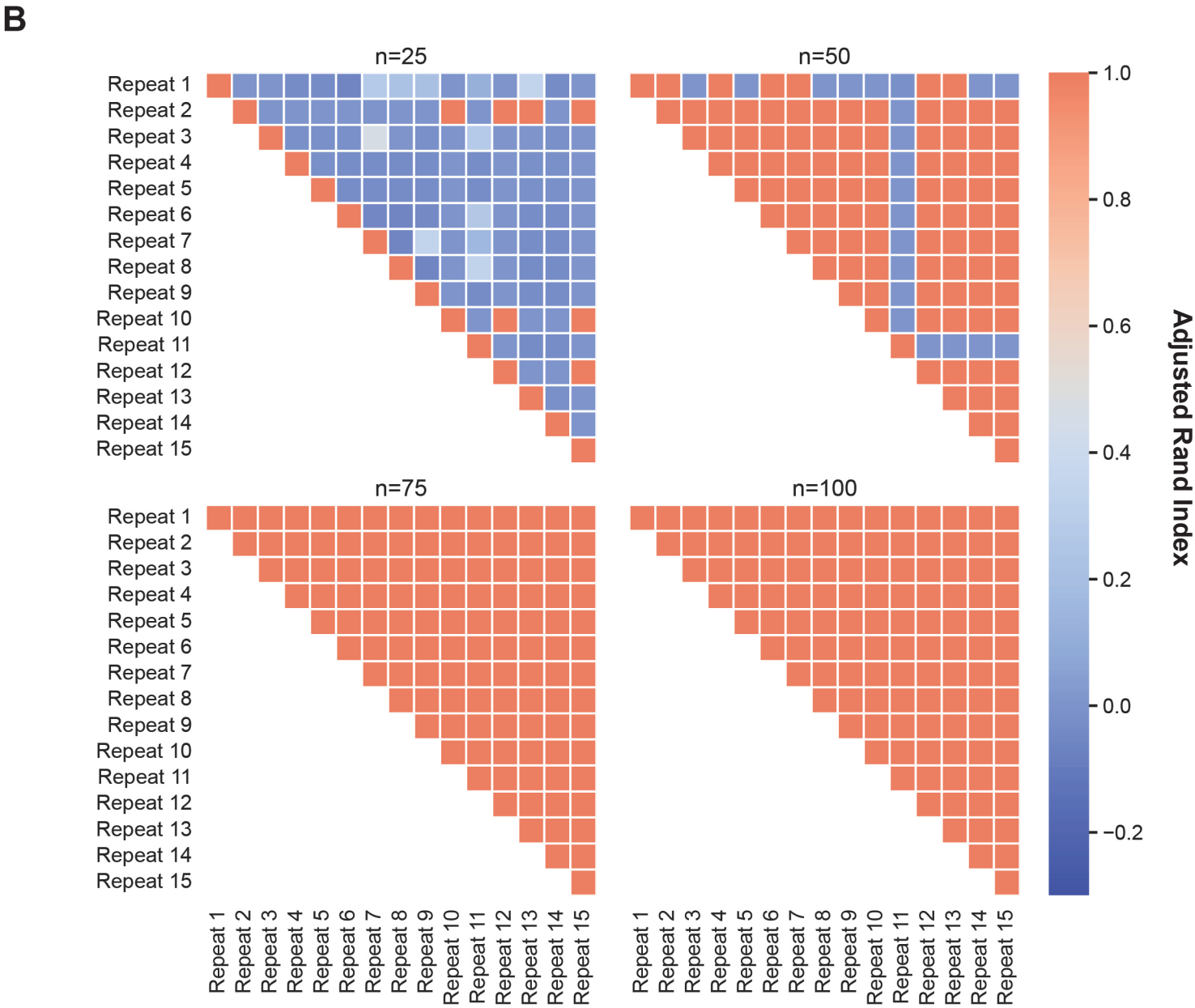

Supplement: Supplemental Figure S6 — Sample size sensitivity analysis.A, phosphosite overlap compared to n=122 and B, adjusted rand index for module BRCA-63 in 15 randomly chosen sets of n = 25, n = 50, n = 75 and n = 100. [file mmc6.pdf]

A

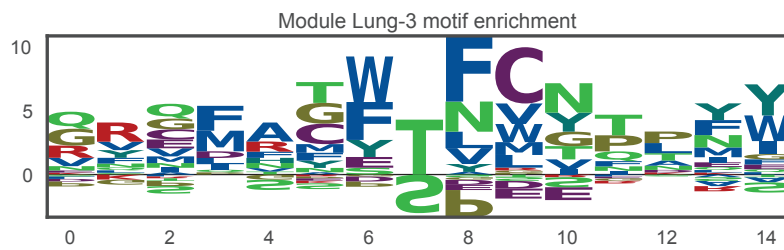

B

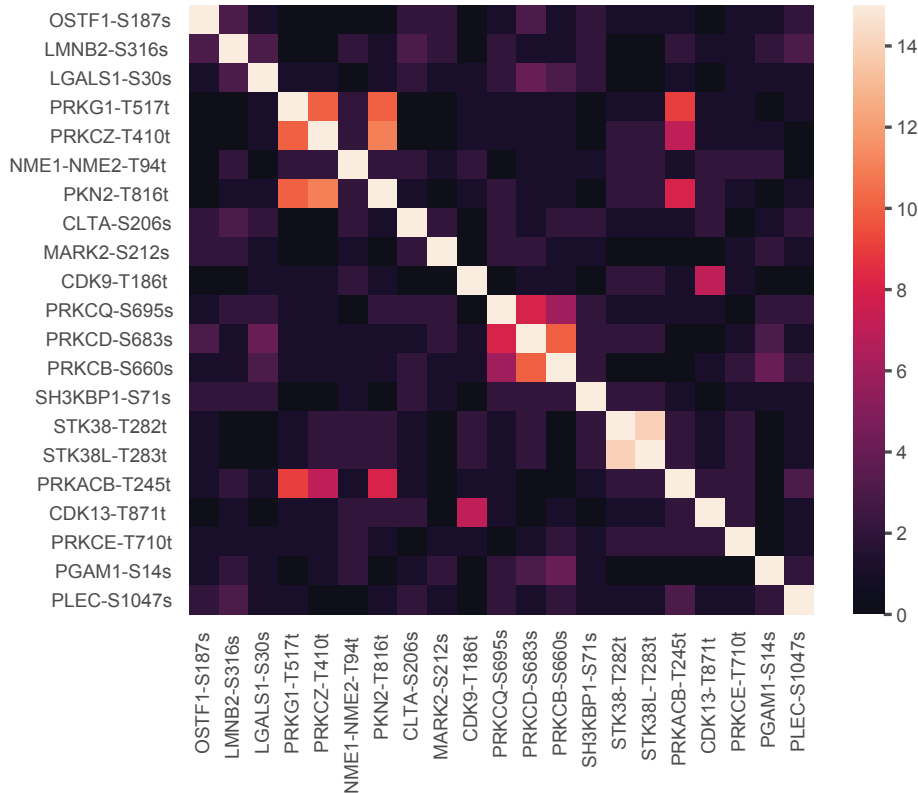

C

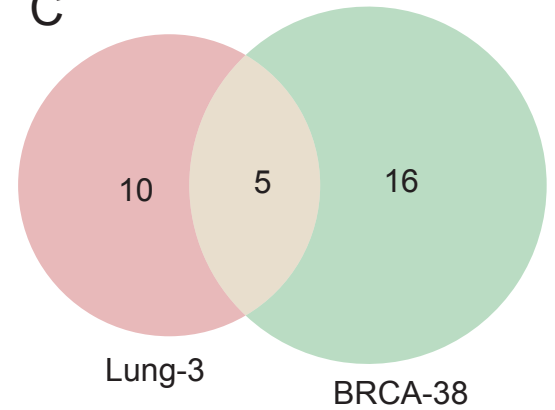

D

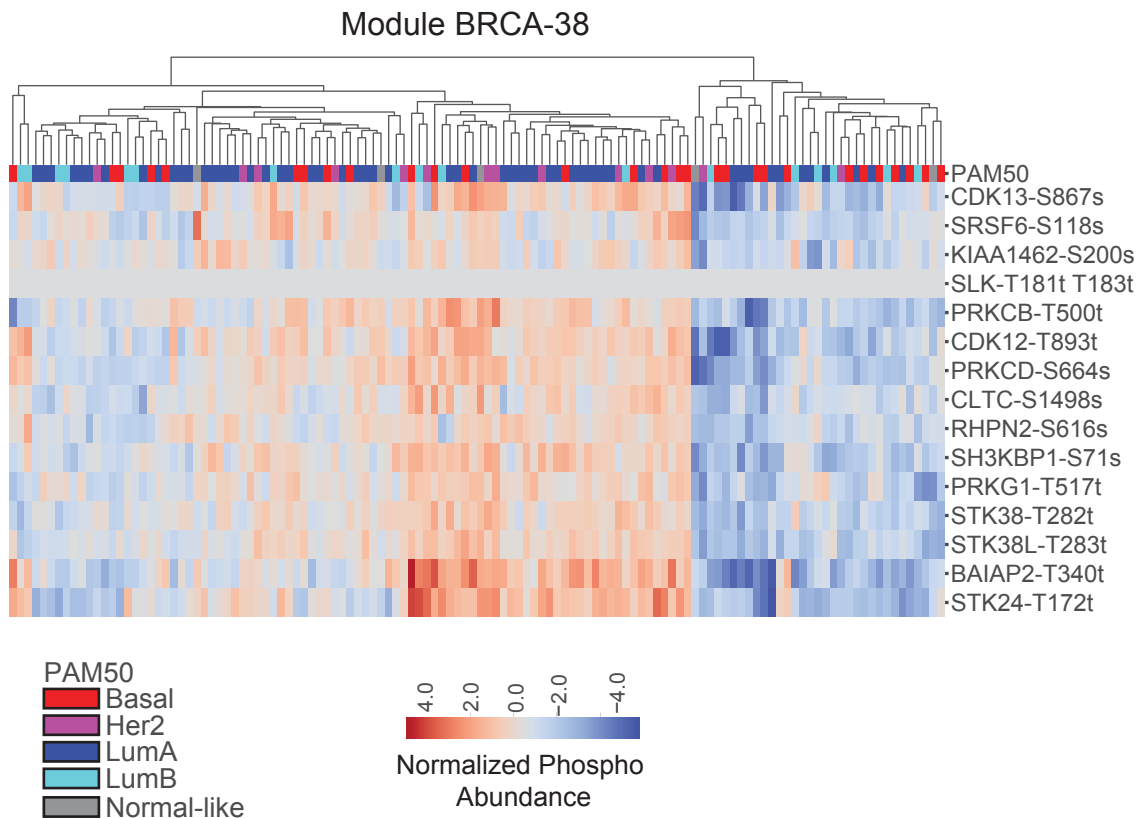

Supplement: Supplemental Figure S7 — Module Lung-3.A, Lung-3 peptide motif of 15 length peptides centered on module phosphosites. B, peptide overlap for 15 length peptides centered on module phosphosites. Color indicates number of overlapping amino acids. C, Venn diagram of phosphosites of Lung-3 and BRCA-38. D, analogous module found in the breast cohort (BRCA-38). [file mmc7.pdf]
